# Comprehensive analysis of the expression of sodium/potassium-ATPase α subunits and prognosis of ovarian serous cystadenocarcinoma

**DOI:** 10.1186/s12935-020-01414-5

**Published:** 2020-07-14

**Authors:** Wei Huang, Yongjian Zhang, Ye Xu, Shaoyou Yang, Bing Li, Lan Huang, Ge Lou

**Affiliations:** 1 Department of Gynecology Oncology, Harbin Medical University Cancer Hospital, Harbin Medical University, Harbin, 150081 Heilongjiang China; 2 Department of Pathology, Harbin Medical University, Harbin, 150081 Heilongjiang China; 3 Department of Medical Oncology, Harbin Medical University Cancer Hospital, Harbin Medical University, Harbin, 150081 Heilongjiang China; 4 Department of Gynecology Oncology, Tumor Hospital of Harbin Medical University, 150 Haping Road, Harbin, 150081 Heilongjiang China; 5 Department of Medical Oncology, Tumor Hospital of Harbin Medical University, Harbin, 150081 Heilongjiang China

**Keywords:** Adenosine triphosphate, Ovary, Cystadenocarcinoma, Gynecology, Gene expression

## Abstract

**Background:**

Ovarian serous cystadenocarcinoma (OSC) is the most common and lethal gynecological cancer in women worldwide; however, biomarkers to diagnose and predict prognosis of OSC remain limited. Therefore, the present study aimed to investigate whether sodium/potassium adenosine triphosphate (Na^+^/K^+^-ATP)ase α-subunits (ATP1As) are helpful diagnostic and prognostic markers of OSC.

**Methods:**

Gene expression data (RNA-Seq) of 376 patients with OSC were downloaded from The Cancer Genome Atlas (TCGA) program database. Additional databases used in our analysis included the Gene Expression Omnibus, International Cancer Genome Consortium, Genotype-Tissue Expression, the Human Protein Atlas, cBioPortal for Cancer Genomics, and Cancer Cell Line Encyclopedia.

**Results:**

The expression levels of ATP1A1 and ATP1A3 were higher in OSC tissues than in normal ovarian tissues, whereas the expression levels of ATP1A2 and ATP1A4 were lower in OSC tissues than in normal ovarian tissues. Overexpression of ATP1A2 was significantly associated with a higher Federation of Gynecology and Obstetrics (FIGO) stage and histological grade. Increased mRNA expression of ATP1A3 was significantly associated with shorter overall survival (OS) and disease-specific survival (DSS) in patients with OSC, whereas higher expression of ATP1A4 was associated with favorable OS and DSS. Multivariate analysis showed that primary therapy outcome, residual tumor, and mRNA expressions of ATP1A3 and ATP1A4 were independent prognostic factors for both OS and DSS in patients with OSC. Moreover, ATP1A1 staining was abundant in tumor tissues. A high expression of ATP1A3 was significantly correlated with poor OS and DSS in the subgroup of patients aged ≥ 60 years and with FIGO stage III, histological grade G3, and TP53 mutation. Mutation frequencies of the ATP1As were 3–5%.

**Conclusions:**

These results indicate that the ATP1A gene family could be potential diagnostic or prognostic markers of OSC. In addition, ATP1As may be effective therapeutic targets in the treatment of OSC.

## Background

Ovarian cancer (OC) is a gynecological malignant tumor associated with high mortality rates and has seriously impaired the health of women worldwide [1]. In nearly 70% of cases, OC has already spread to the pelvic and abdominal organs by the time of the initial diagnosis due to the asymptomatic nature of this cancer in early stages and the limited predictive biomarkers [2]. In addition, the prognosis of advanced OC is extremely poor, with a 5-year survival rate of only 30% [3]. Ovarian serous cystadenocarcinoma (OSC) accounts for nearly 40–60% of epithelial OCs and is the most common and most lethal pathological type of OCs [4]. Therefore, identification of biomarkers for early diagnosis and prognosis prediction of OSC remains a crucial clinical challenge of important clinical significance.

Sodium/potassium adenosine triphosphate (Na^+^/K^+^-ATP)ase (ATP1A) is a type of ubiquitous transmembrane protein expressed widely in mammalian cells, and it plays an important role in maintaining the electrochemical gradient across the cell membrane [5]. It mainly comprises a catalytic α subunit and a regulatory β subunit. The α subunit has four isoforms: α1 (ATP1A1), α2 (ATP1A2), α3 (ATP1A3), and α4 (ATP1A4). The β subunit has three isoforms: β1 (ATP1B1), β2 (ATP1B2), and β3 (ATP1B3) [5]. Different combinations of α and β subunits can form different subtypes of Na^+^/K^+^-ATPase, and their distribution is tissue specific.

Previous studies have reported abnormal expressions in some members of the ATP1A gene family with prognostic value [6–10]. It is reported that ATP1A1 is overexpressed in clinical specimens and cell lines of non-small cell lung cancer. Moreover, knockdown of ATP1A1 impaired the proliferation and migration of tumor cells [6]. Elevated levels of ATP1A1 and ATP1A3 have been reported in patients with medulloblastoma [7]. Overexpressed ATP1B3 in gastric cancer promotes tumor proliferation, invasion, anti-apoptosis and cell-cycle arrest. Mechanically, ATP1B3 promotes the malignant progression of gastric cancer via the phosphatidylinositol 3-kinase (PI3K)/protein kinase B (AKT) signaling pathway [8]. High expression of ATP1B2 has been associated with poor prognosis of patients with glioblastoma, and downregulation of ATP1B2 can inhibit cell growth and induce cell apoptosis and cell-cycle arrest [9]. In addition, the expression of ATP1B2 in glioblastoma stem-like cells was significantly increased, suggesting its association with the stemness of tumor cells [9]. A previous study has also found that the expression of ATP1As and ATP1Bs can be used as predictors of clinical outcomes in bladder cancer [10]. Nevertheless, the role of distinct ATP1A gene family members in the development and progression of OSC has not been fully elucidated.

In the present study, we comprehensively analyzed the expression profiles of patients with OSC based on The Cancer Genome Atlas (TCGA) database and analyzed the correlation between the ATP1A gene family and clinically related information, including prognosis information. In addition, we used the OC clinical data in the Gene Expression Omnibus (GEO) database and International Cancer Genome Consortium (ICGC) database for further verification and analysis. We also interpreted the results of immunohistochemistry based on gene expression in OSC and normal tissue samples and validated them in patient samples. Finally, we analyzed the mRNA expression of this gene family in pan-cancer tissues and cell lines. Our results therefore suggest that the ATP1A gene family members as potential diagnostic or prognostic markers of OSC.

## Materials and methods

### RNA-sequencing (RNA-Seq) patient data and bioinformatics analysis

We collected RNA-Seq data and the corresponding clinical information of patients with OSC from the OC project of the TCGA database [11]. A total of 376 cases were obtained from the TCGA cohort after discarding cases without clinical information. The RNA-Seq data of level 3 HTSeq fragments per kilobase per million were converted to the transcripts per million reads format for further analysis. Unavailable or unknown clinical information was considered missing values. The RNA-Seq data of normal ovarian tissue samples were downloaded from the standardized Genotype-Tissue Expression (GTEx) [12] database on the Xena platform (https://xenabrowser.net/) [13] as control samples. In addition, we downloaded the expression profile data set and clinical information data of OC in the Ovarian Cancer-AU (OV-AU) dataset from the ICGC (https://dcc.icgc.org/) database. The samples included 81 OC samples and 30 normal control samples. Clinical features of patients with OC from the ICGC database are shown in Additional file 1: Table S1. Based on the GPL570 platform ([HG-U133_Plus_2] Affymetrix Human Genome U133 Plus 2.0 Array), we used the R language GEO query package [14] to download and analyze the OC expression profile data set GSE26193 [15] from the GEO database. *Homo sapiens* was selected as the species, and a total of 107 OC samples were obtained. Clinical features of patients with OC from GSE26193 are shown in Additional file 1: Table S2. We used the built-in function of limma package [16] to correct the expression profile data matrix of OC in the ICGC and GEO datasets; extract the expression matrixes of ATP1A1, ATP1A2, ATP1A3, and ATP1A4; and analyze the clinical data.

### Human Protein Atlas (HPA)

HPA (https://www.proteinatlas.org) is a website based on tumor immunohistochemical expression data and contains nearly 20 of the most common types of cancer. Each tumor type includes 12 individual tumors [17]. Users can identify tumor-specific protein expression patterns that are differentially expressed in a given type of tumor. In the present study, direct comparison of protein expression of different ATP1A gene family members between human normal and OSC tissue samples was performed by analysis of immunohistochemistry images.

### Broad Institute Cancer Cell Line Encyclopedia (CCLE)

The Broad CCLE (https://portals.broadinstitute.org/ccle) is a tumor genomics research project led by the Broad Institute, which collates the data of more than 1000 tumor cell lines [18]. In the present study, we directly compared the mRNA expression of the ATP1A gene family members in different cancer cell lines.

### cBioPortal

cBioPortal (www.cbioportal.org), an online open access website, is a resource for exploring, visualizing, and analyzing multidimensional cancer genomics data [19]. In the present study, we analyzed the mutation frequency of the four members of the ATP1A gene family based on the cBioPortal database.

### Clinical samples and immunohistochemistry staining

The cancer and paracancerous tissues of six patients with OSC were embedded in paraffin and cut into tissue sections. These sections were placed in xylene to dewax and rehydrated with gradient alcohol. EDTA (pH 9.0) was used for antigen recovery, and 3% hydrogen peroxide was used to block endogenous peroxidase. The primary antibodies were added, and the sections were incubated at 4 °C overnight. Then, the sections were incubated with secondary antibodies and stained with 3′-diaminobenzidine. The primary antibodies used were ATP1A1 (1: 200; Proteintech; 14418-1-AP), ATP1A2 (1: 200; Proteintech; 16836-1-AP), and ATP1A3 (1: 200; Proteintech; 10868-1-AP). All patients received no treatment before surgery. The study was approved by Ethics Committee of the Cancer Hospital of Harbin Medical University (Harbin, China), and all patients signed an informed consent form.

### Statistical methods

Statistical analysis was performed using the R software (version 3.6.2). Wilcoxon rank sum test was used to analyze the expression of the ATP1A gene family members in normal and cancer tissues. Receiver operating characteristic (ROC) curve was used to analyze the sensitivity and specificity of the ATP1A gene family members in predicting the prognosis of OSC and OC. Kruskal–Wallis test and Wilcoxon signed-rank test were used to analyze the relationship between the clinicopathological features and the expression of ATP1A genes. Cox regression analysis or Kaplan–Meier method was used to evaluate the prognostic value of ATP1A genes. In Cox regression analysis, the variables with *p* < 0.1 in univariate Cox regression were included in multivariate Cox regression. A two-sided *p* value of < 0.05 was considered to be statistically significant.

## Results

### Patient characteristics

As shown in Table 1, 376 cases of primary OSC with clinical and gene expression data were downloaded from the TCGA database. Among these, 178 patients (47.3%) were aged > 60 years. Federation of Gynecology and Obstetrics (FIGO) stage I disease was found in 1 patient (0.3%), stage II in 22 (5.9%), stage III in 293 (78.6%), and stage IV in 57 (15.3%). The histological grades, G1, G2, G3, and G4 were found in 0.3%, 11.5%, 88.0%, and 0.3% of the patients, respectively. The cancer status included 71 without (21.3%) and 262 with (78.7%) tumors. The patients were divided into four groups according to the primary therapy outcome: progressive disease (PD), 27 (8.9%); partial response (PR), 43 (14.1%); stable disease (SD), 22 (7.2%); and complete response (CR), 213 (69.8%). Residual tumors were found in 267 of the 376 total cases (23.5%), lymphatic invasion in 100 of 148 cases (67.6%), and venous invasion in 63 of 103 cases (61.2%). Lesions occurred in the bilateral ovaries of 253 cases. Mutations of TP53 and breast cancer susceptibility genes (BRCA) were found in 248 and 25 cases, respectively. Of the patients in this cohort, 61.2% finally succumbed to OSC (Table 1).

**Table 1:** Clinical characteristics of 376 ovarian cancer patients in TCGA database

Characteristics	Total
	N = 376 (%)
Age	
< 60	198 (52.7)
≥ 60	178 (47.3)
FIGO stage	
Stage I	1 (0.3)
Stage II	22 (5.9)
Stage III	293 (78.6)
Stage IV	57 (15.3)
Histologic grade	
G1	1 (0.3)
G2	42 (11.5)
G3	322 (88.0)
G4	1 (0.3)
Recurrence	
No	176 (46.8)
Yes	200 (53.2)
Tumor status	
Tumor free	71 (21.3)
With tumor	262 (78.7)
Primary therapy outcome	
PD	27 (8.9)
SD	22 (7.2)
PR	43 (14.1)
CR	213 (69.8)
Tumor residual	
NRD	66 (19.8)
RD	267 (80.2)
Lymphatic invasion	
No	48 (32.4)
Yes	100 (67.6)
Venous invasion	
No	40 (38.8)
Yes	63 (61.2)
Anatomic subdivision	
Unilateral	101 (28.5)
Bilateral	253 (71.5)
TP53 mutation	
No	26 (9.5)
Yes	248 (90.5)
BRCA mutation	
No	249 (90.9)
Yes	25 (9.1)
OS event	
Alived	146 (38.8)
Dead	230 (61.2)

*PD* progression disease, *SD* stable disease, *PR* partial response, *CR* complete response, *NRD* no residual disease, *RD* residual disease, *OS* overall survival

We compared the hazard ratios (HR) with 95% confidence interval (CI) ranges among patient information parameters. Univariate analysis identified primary therapy outcome (*p *< 0.001), age (*p *= 0.032), residual tumor (*p *< 0.001), ATP1A3 (*p *< 0.001), and ATP1A4 (*p *= 0.03) as prognostic factors for OS (Table 2). Multivariate analysis showed that primary therapy outcome (*p *< 0.001), residual tumor (*p *= 0.004), ATP1A2 (*p *= 0.006), ATP1A3 (*p *< 0.001), and ATP1A4 (*p *< 0.001) were independent risk factors for OS (Table 2).

**Table 2:** Univariate and multivariate analysis of clinical variables and ATP1As for OS

Characteristics	Univariate analysis		Multivariate analysis	
	Hazard ratio (95% CI)	*p*-value	Hazard ratio (95% CI)	*p*-value
FIGO stage (stage I–II vs. stage III–IV)	2.085 (0.925–4.699)	0.076	1.638 (0.392–6.854)	0.499
Histologic grade (G1–2 vs. G3–4)	1.194 (0.797–1.789)	0.389		
Primary therapy outcome (PR-CR vs. SD-PD)	0.306 (0.207–0.451)	*< 0.001*	0.247 (0.161–0.38)	*< 0.001*
Age (< 60 vs. ≥ 60)	1.329 (1.025–1.722)	*0.032*	1.305 (0.948–1.794)	0.102
Tumor residual (NRD vs. RD)	2.302 (1.479–3.583)	*< 0.001*	2.149 (1.285–3.594)	*0.004*
Lymphatic invasion (no vs. yes)	1.422 (0.839–2.411)	0.191		
Venous invasion (no vs. yes)	0.905 (0.487–1.683)	0.753		
Anatomic subdivision (unilateral vs. bilateral)	1.041 (0.768–1.41)	0.798		
TP53 mutation (no vs. yes)	0.692 (0.423–1.132)	0.143		
BRCA mutation (no vs. yes)	0.65 (0.361–1.171)	0.152		
ATP1A1 (low vs. high)	1.148 (0.885–1.49)	0.298		
ATP1A2 (low vs. high)	1.257 (0.968–1.632)	0.086	1.564 (1.134–2.157)	*0.006*
ATP1A3 (low vs. high)	1.6 (1.232–2.077)	*< 0.001*	1.932 (1.397–2.673)	*< 0.001*
ATP1A4 (low vs. high)	0.748 (0.575–0.972)	*0.03*	0.555 (0.4–0.771)	*< 0.001*

Univariate analysis identified primary therapy outcome (*p *< 0.001), residual tumor (*p *< 0.001), ATP1A3 (*p *= 0.002), and ATP1A4 (*p *= 0.023) as prognostic factors for DSS (Table 3). Multivariate analysis showed that primary therapy outcome (*p *< 0.001), residual tumor (*p *= 0.001), ATP1A3 (*p *= 0.006), and ATP1A4 (*p *= 0.017) were independent risk factors for DSS (Table 3).

**Table 3:** Univariate and multivariate analysis of clinical variables and ATP1As for DSS

Characteristics	Univariate analysis		Multivariate analysis	
	Hazard ratio (95% CI)	*p*-value	Hazard ratio (95% CI)	*p*-value
FIGO stage (stage I–II vs. stage III–IV)	2.244 (0.922–5.462)	0.075	1.092 (0.257–4.639)	0.905
Histologic grade (G1–2 vs. G3–4)	1.313 (0.833–2.07)	0.24		
Primary therapy outcome (SD-PD vs. PR-CR)	0.299 (0.201–0.443)	*< 0.001*	0.296 (0.169–0.517)	*< 0.001*
Age (< 60 vs. ≥ 60)	1.248 (0.944–1.65)	0.12		
Tumor residual (NRD vs. RD)	2.559 (1.572–4.166)	*< 0.001*	2.991 (1.526–5.86)	*0.001*
Lymphatic invasion (no vs. yes)	1.407 (0.816–2.425)	0.219		
Venous invasion (no vs. yes)	0.846 (0.45–1.591)	0.604		
Anatomic subdivision (unilateral vs. bilateral)	1.034 (0.747–1.431)	0.841		
TP53 mutation (no vs. yes)	0.643 (0.386–1.07)	0.089	1.026 (0.565–1.861)	0.934
BRCA mutation (no vs. yes)	0.596 (0.313–1.134)	0.115		
ATP1A1 (low vs. high)	1.135 (0.857–1.501)	0.377		
ATP1A2 (low vs. high)	1.304 (0.984–1.727)	0.064	1.413 (0.958–2.085)	0.082
ATP1A3 (low vs. high)	1.572 (1.188–2.081)	*0.002*	1.723 (1.169–2.54)	*0.006*
ATP1A4 (low vs. high)	0.721 (0.544–0.956)	*0.023*	0.611 (0.408–0.916)	*0.017*

### Correlation between expression of ATP1A genes and the clinical characteristics

**Fig. 1:**
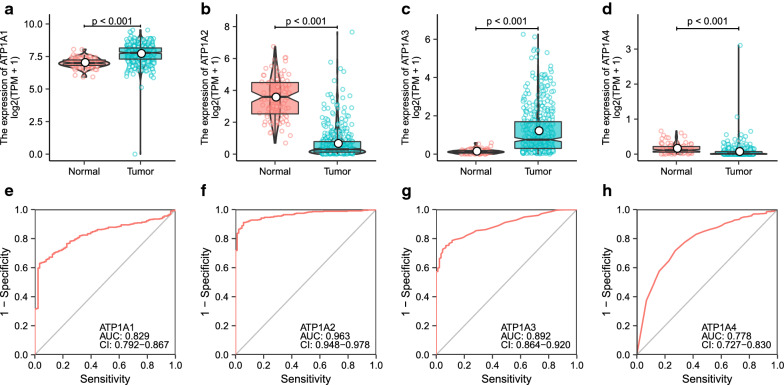
Expression of ATP1A gene family members in ovarian serous cystadenocarcinoma (OSC) and its diagnostic value. **a**–**d** Differential expression analysis of ATP1A gene family members between OSC samples based on the TCGA database and normal ovarian samples based on the GTEx database. **e**–**h** Receiver-operating characteristic (ROC) curves for ATP1A gene family members for diagnosis of OSC. (*AUC* area under curve, *CI* confidence interval, *N* normal, *T* tumor)

The expression of ATP1A gene family members in OSC and normal tissues were significantly different. ATP1A1 and ATP1A3 were highly expressed in tumor tissues, whereas ATP1A2 and ATP1A4 were highly expressed in normal tissues (all *p* < 0.001) (Fig. 1a–d). Based on the OV-AU dataset, we found that ATP1A2 was highly expressed in OC tissues (p < 0.001), whereas ATP1A3 was highly expressed in normal tissues (*p* = 0.002). In addition, there was no difference in the expression of ATP1A1 and ATP1A4 between OC and normal tissues (Additional file 2: Figure S1A–D). To evaluate the expression of the ATP1A gene family members in human pan-cancers, the RNA-Seq data from the TCGA and GTEx databases were further analyzed. Differential expression between the tumor and adjacent normal tissues for ATP1A1-4 across all TCGA tumors is shown in Additional file 3: Figure S2. ATP1A1 expression was significantly higher in cholangiocarcinoma, skin cutaneous melanoma and thymoma (THYM) (all *p* < 0.001) than in the adjacent normal tissues. ATP1A2 expression was significantly lower in breast invasive carcinoma, bladder urothelial carcinoma and colon adenocarcinoma (all *p* < 0.001) than in the adjacent normal tissues. ATP1A3 expression was significantly higher in adrenocortical carcinoma (*p* < 0.001), THYM (*p* < 0.001) and pheochromocytoma and paraganglioma (*p* < 0.01) than in the adjacent normal tissues. ATP1A4 expression was significantly higher in BRCA, lymphoid neoplasm diffuse large B-cell lymphoma and lung squamous cell carcinoma (all *p* < 0.001) (Additional file 3: Figure S2). In addition, the mRNA expression of the ATP1A gene family members in pan-cancer cell lines was also analyzed based on the CCLE database (Additional file 4: Figure S3). As observed, ATP1A1 was highly expressed in Ewing’s-sarcoma, melanoma, and colorectal carcinoma, whereas ATP1A3 was highly expressed in neuroblastoma, Burkitt lymphoma, and T-lymphocytic leukemia. Conversely, the expression of ATP1A2 and ATP1A4 was lower in most tumor cell lines (Additional file 4: Figure S3).

Next, we analyzed the sensitivity and specificity of the ATP1A gene family members in predicting the diagnosis of OSC by ROC curve. The area under the curve (AUC) for ATP1A1, ATP1A2, ATP1A3, and ATP1A4 were 0.829, 0.963, 0.892 and 0.778, respectively (Fig. 1e–h). The results from the OV-AU dataset are consistent with those from the TCGA database. The AUC for ATP1A1, ATP1A2, ATP1A3, and ATP1A4 in patients with OC were 0.555, 0.729, 0.704, and 0.507, respectively (Additional file 2: Figure S1E–H). These results suggested that the ATP1A gene family members are potential diagnostic biomarkers for both OSC and OC.

**Fig. 2:**
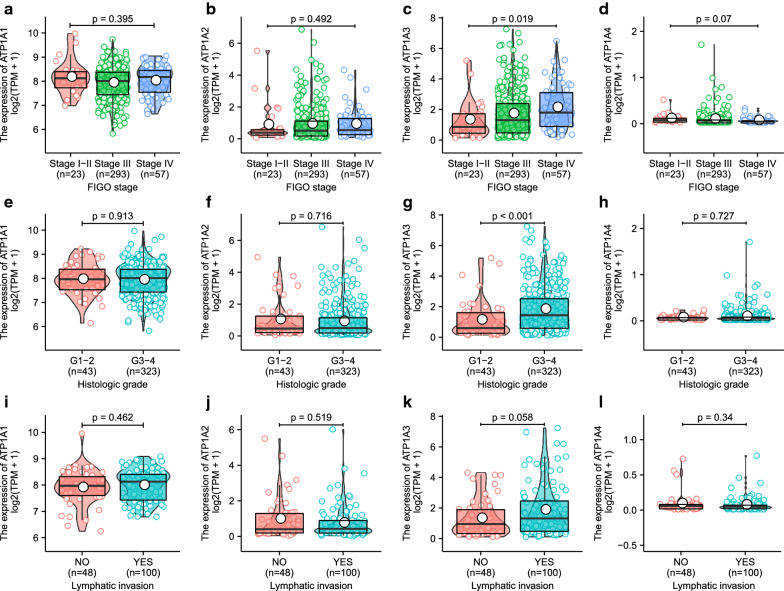
Analysis of ATP1A gene family member expression and clinical correlations in ovarian serous cystadenocarcinoma (OSC). Correlation between FIGO stage and ATP1A1 (**a**), ATP1A2 (**b**), ATP1A3 (**c**), and ATP1A4 (**d**). Correlation between histological grade and ATP1A1 (**e**), ATP1A2 (**f**), ATP1A3 (**g**), and ATP1A4 (**h**). **i**–**l** Correlation between lymphatic invasion and ATP1A1 (**i**), ATP1A2 (**j**), ATP1A3 (**k**), and ATP1A4 (**l**)

Moreover, we analyzed the correlation between the expression of the ATP1A gene family members and the clinical features, as revealed by the Kruskal–Wallis test and Wilcoxon signed-rank test. Significant correlation was noted between ATP1A3 (*p* = 0.019) and FIGO stage, but no significant correlations were found between ATP1A1 (*p* = 0.395), ATP1A2 (*p* = 0.492), ATP1A4 (*p* = 0.07), and FIGO stage (Fig. 2a–d). In addition, ATP1A3 (*p* < 0.001) was significantly associated with histological grade, whereas ATP1A1 (*p* = 0.913), ATP1A2 (*p* = 0.716), and ATP1A4 (*p* = 0.727) were not (Fig. 2e–h). Although no correlation was found between ATP1As and lymphatic invasion, the *p* value of ATP1A3 was close to 0.05, suggesting that the lack of significance may be due to a limitation of the number of patients (Fig. 2i–l). By analyzing the GSE26193 dataset, no significant correlations were found between ATP1A1 (*p* = 0.21), ATP1A2 (*p* = 0.35), ATP1A3 (*p* = 0.9), ATP1A4 (*p* = 0.59), and FIGO stage (Additional file 5: Figure S4A–D). In addition, we found a significant correlation between ATP1A1 and histological grade (*p* = 0.008), whereas ATP1A2 (*p* = 0.87), ATP1A3 (*p* = 0.882), and ATP1A4 (*p* = 0.453) were not (Additional file 5: Figure S4E–H).

**Fig. 3:**
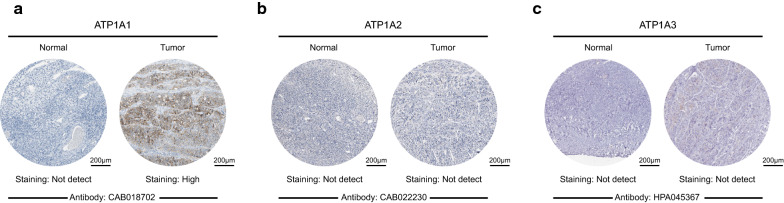
Representative immunohistochemistry images of ATP1A gene family members in OSC and normal ovarian tissues (Human Protein Atlas). **a** ATP1A1 expression was not detected in normal ovarian tissues, whereas its high expression was detected in OSC tissues. **b**, **c** ATP1A2 and ATP1A3 expression was not detected both in normal and OSC tissues

Based on these mRNA expression patterns of the ATP1A gene family members in OSC, we next explored the protein expression patterns of ATP1As in OSC using HPA. As shown in Fig. 3, ATP1A1 was highly expressed in the OSC tissue than in the normal ovarian tissues (Fig. 3a). In addition, the protein expression of ATP1A2 and ATP1A3 was low in both normal and OSC tissues (Fig. 3b, c). A lack of protein expression patterns of ATP1A4 genes in the database at present precluded our analysis of this protein.

### Prognostic value of mRNA expression of ATP1A gene family members in patients with OSC

**Fig. 4:**
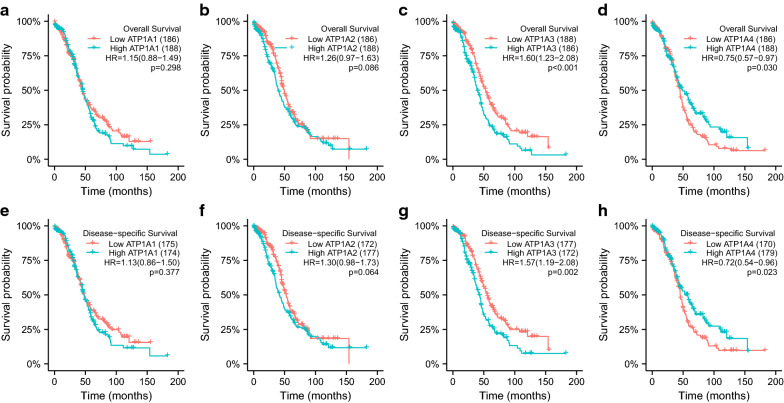
Prognostic analysis of the ATP1A gene family members. **a**–**d** Effects of ATP1A gene family members on overall survival (OS). **e**–**h** Effects of ATP1A gene family members on disease-specific survival (DSS). (*p* < 0.05 was considered statistically significant)

We used a Kaplan–Meier plotter (http://kmplot.com/analysis/) to analyze the prognostic values of the mRNA expression of ATP1A genes in both patients with OSC and OC patients. First, we analyzed the relationship between the mRNA expressions of distinct ATP1A gene family members and the prognoses of patients with OSC. As shown in Fig. 4, for OS and DSS, a higher mRNA expression of ATP1A3 (HR = 1.60, CI 1.23–2.08, *p* < 0.001; and HR = 1.57, CI 1.19–2.08, *p* = 0.002, respectively) (Fig. 4c, d) and ATP1A4 (HR = 0.75, CI 0.57–0.97, *p* = 0.030; and HR = 0.72, CI 0.54–0.96, p = 0.023, respectively) (Fig. 4g, h) were significantly associated with shorter OS and DSS in patients with OSC. However, the mRNA expression of neither ATP1A1 (HR = 1.15, CI 0.88–1.49, *p* = 0.298; and HR = 1.13, CI 0.86–1.50, *p* = 0.377, respectively) (Fig. 4a, b) nor ATP1A2 (HR = 1.26, CI 0.97–1.63, *p* = 0.086; and HR = 1.30, CI 0.98–1.73, *p* = 0.064, respectively) (Fig. 4e, f) showed any correlation with OS or DSS in patients with OSC. Moreover, the results of survival analysis from the GSE26193 datasets are showed in Additional file 6: Figure S5. It was found that higher mRNA expressions of ATP1A3 (HR = 1.86, CI 1.13–3.07, *p* = 0.014; and HR = 1.74, CI 1.07–2.81, *p* = 0.023, respectively) and ATP1A4 (HR = 1.25, CI 1.02–1.54, *p* = 0.029; and HR = 2.17, CI 1.25–3.77, p = 0.0047, respectively) were significantly associated with shorter OS and progression-free survival (PFS) in patients with OC. In contrast, a higher mRNA expression of ATP1A1 was associated with longer OS or PFS (HR = 0.58, CI 0.33–1.01, *p* = 0.053; and HR = 0.51, CI 0.3–0.87, *p* = 0.011, respectively) (Additional file 6: Figure S5). These results suggested that the mRNA expressions of ATP1A3 and ATP1A4 were closely related to the prognosis of OC. They may be used as potential biomarkers to predict the survival of patients.

**Fig. 5:**
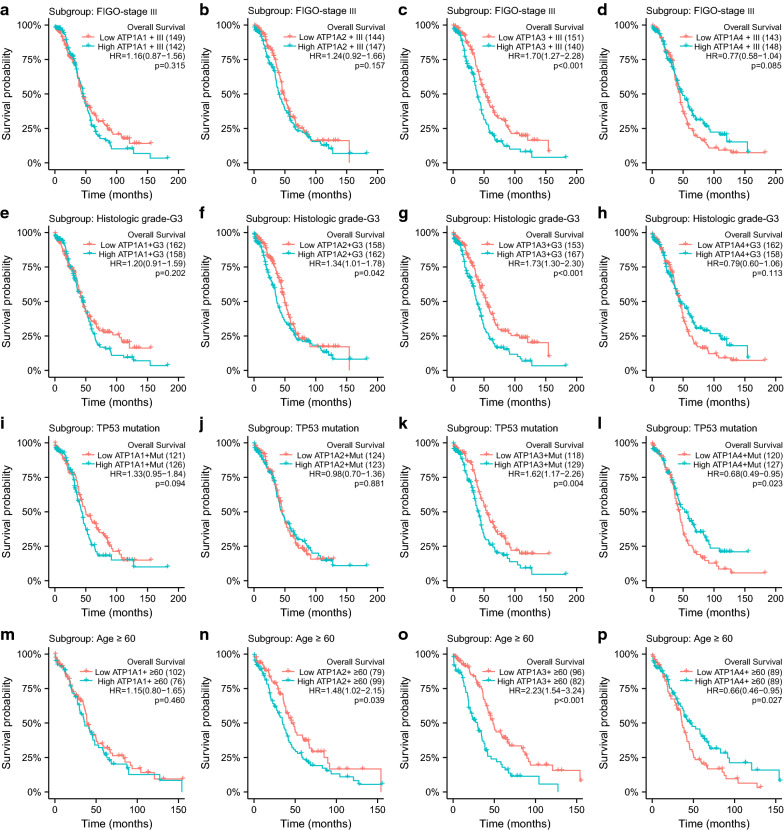
Prognostic analysis of the ATP1A gene family members in subgroups for overall survival. Prognostic analysis of ATP1A1 (**a**), ATP1A2 (**b**), ATP1A3 (**c**), and ATP1A4 (**d**) for FIGO stage III. Prognostic analysis of ATP1A1 (**e**), ATP1A2 (**f**), ATP1A3 (**g**), and ATP1A4 (**h**) for histological grade G3. Prognostic analysis of ATP1A1 (**i**), ATP1A2 (**j**), ATP1A3 (**k**), and ATP1A4 (**l**) for TP53 mutation. Prognostic analysis of ATP1A1 (**m**), ATP1A2 (**n**), ATP1A3 (**o**), and ATP1A4 (**p**) for age ≥ 60. (*p* < 0.05 was considered statistically significant)

Secondly, we analyzed the effects of distinct ATP1A gene family member expression on patients’ prognosis in the subgroups of FIGO stage III, histological grade G3, TP53 mutation, and age ≥ 60 years. The results showed that in patients with FIGO stage III, no significant correlation was found in ATP1A1 (HR = 1.16, CI 0.87–1.56, *p* = 0.315), ATP1A2 (HR = 1.24, CI 0.92–1.66, *p* = 0.157), and ATP1A4 (HR = 0.77, CI 0.58–1.04, *p* = 0.085), whereas the high expression of ATP1A3 (HR = 1.70, CI 1.27–2.28, *p* < 0.001) was significantly associated with shorter OS (Fig. 5a–d). Higher mRNA expressions of ATP1A2 (HR = 1.34, CI 1.01–1.78, *p* = 0.042) and ATP1A3 (HR = 0.75, CI 1.30–2.30, *p* < 0.001) were significantly associated with poor OS in patients with histological grade G3, whereas no such association was noted for ATP1A1 (HR = 1.20, CI 0.91–1.59, *p* = 0.202) and ATP1A4 (HR = 0.79, CI 0.60–1.06, *p* = 0.113) were not (Fig. 5e–h). In addition, the high expression of ATP1A3 (HR = 1.62, CI 1.17–2.26, *p* = 0.004) was significantly associated with decreased OS in patients with TP53 mutation, but no correlation was observed with ATP1A1 (HR = 1.33, CI 0.95–1.84, *p* = 0.094), ATP1A2 (HR = 0.98, CI 0.70–1.36, *p* = 0.881) and ATP1A4 (HR = 0.68, CI 0.49–0.95, *p* = 0.023) (Fig. 5i–l). Furthermore, we found that the high expression of ATP1A2 (HR = 1.48, CI 1.02–2.15, *p* = 0.039) and ATP1A3 (HR = 2.23, CI 1.54–3.24, *p* < 0.001) was significantly associated with poor OS in patients aged ≥ 60 years old, whereas no such correlation was observed with ATP1A1 (HR = 1.15, CI 0.80–1.65, *p* = 0.460). Conversely, we found that the high expression of ATP1A4 (HR = 0.66, CI 0.46–0.95, *p* = 0.027) was correlated with better OS (Fig. 5m–p). Finally, the effects of ATP1A gene family members on DSS in patients in subgroups are shown in Additional file 7: Figure S6.

**Fig. 6:**
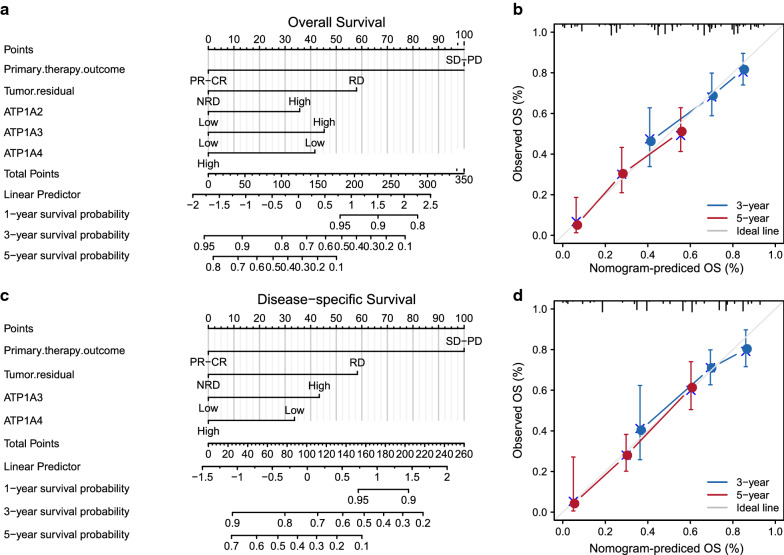
Multivariate analysis of clinical characteristics based on ATP1A gene family member expression. **a** A nomogram for predicting overall survival (OS) in patients with ovarian serous cystadenocarcinoma (OSC) patients in the training cohort based on clinicopathological parameters. **b** The calibration image shows the OS analysis and prediction efficiency of the multivariate Cox model. **c** A nomogram for predicting disease-specific survival (DSS) in patients with OSC in the training cohort based on clinicopathological parameters. **d** The calibration image shows the DSS analysis and prediction efficiency of the multivariate Cox model

Based on the results of multivariate analysis, a genomic-clinical nomogram, including primary tumor therapy outcome, residual tumor, ATP1A2, ATP1A3, and ATP1A4, was established to predict the 1-, 3-, and 5-year OS and DSS in patients with OC (Fig. 6a, c). The calibration curves of the nomogram for predicting these survival times indicated that it performed well (Fig. 6b, d).

**Fig. 7:**
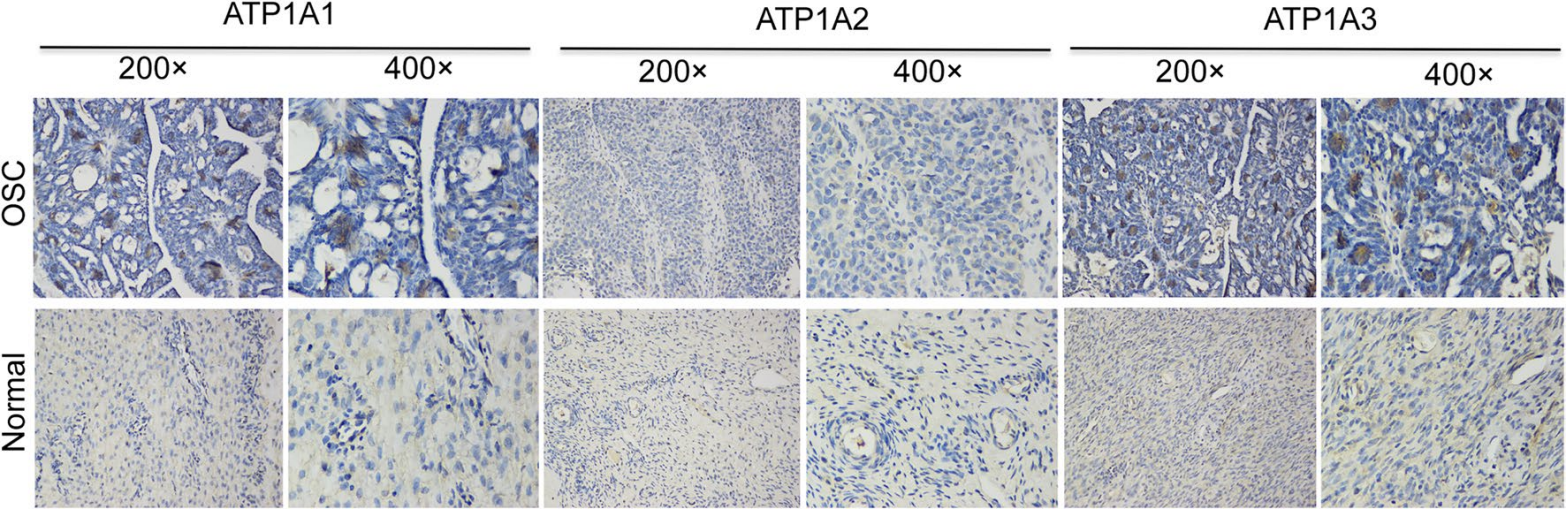
Representative images of ATP1A gene family member expression in OSC and paracancerous tissue samples

To further validate our data, we tested the protein expression of the ATP1A gene family members (including ATP1A1, ATP1A2, and ATP1A3) in cancer and paracancerous tissue samples of six patients with OSC. Among these six patients, one had FIGO stage IIIA and the other five had stage IIIC. No patient had lymph node metastasis. Our results showed that ATP1A1 was positively expressed in three OSC samples and negatively expressed in all the paracancerous samples; ATP1A2 was negatively expressed in both the OSC and paracancerous samples; ATP1A3 was positively expressed in five OSC samples and three paracancerous samples. Images of ATP1A gene family member expression in tumor and paracancerous tissues are presented in Fig. 7.

### Mutation frequency of ATP1A gene family members

**Fig. 8:**
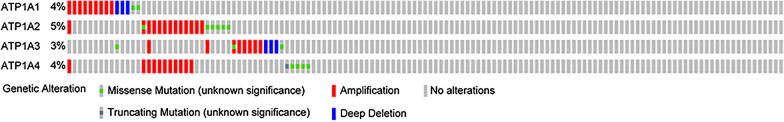
Mutation frequency of ATP1A gene family members in ovarian serous cystadenocarcinoma. The mutation frequencies of ATP1A1, ATP1A2, ATP1A3, and ATP1A4 in ovarian serous cystadenocarcinoma were 4%, 5%, 3%, and 4%, respectively

Based on the cBioPortal database, we explored the mutation frequency of the ATP1A gene family members. The mutation rates of ATP1A1, ATP1A2, ATP1A3, and ATP1A4 were 4%, 5%, 3%, and 4%, respectively (Fig. 8).

## Discussion

To date, the clinical prognosis of patients with OSC remains unsatisfactory due to the lack of predictive biomarkers for early diagnosis and effective treatment targets. Therefore, it is important to understand the molecular aberration underlying the occurrence and development of OSC to identify new molecular markers for early diagnosis, targeted treatment and prognosis evaluation.

In recent years, accumulated evidence has suggested the involvement of Na^+^/K^+^-ATPase in tumor occurrence and development. Alterations in Na^+^/K^+^-ATPase subunits have been observed in various tumors [6, 20]. As an important component of Na^+^/K^+^-ATPase, α subunits play key roles in catalysis. Previous studies have found that overexpression of ATP1A1 is associated with the occurrence and development of several cancers such as breast cancer, non-small cell lung cancer, renal clear–cell carcinoma, and glioma [21–23]. However, the clinical relevance of the expression of ATP1A gene family members in OSC is still not clear.

Considering that genes have been widely used as biomarkers to predict the risk of recurrence of malignant tumors, herein, we explored the diagnostic and predictive values of the ATP1A gene family members in OSC by using a series of bioinformatics methods. We used the TCGA and GTEx databases to explore the differential mRNA expression of the four ATP1A gene family members in OSC and normal ovarian tissues. The results showed that the expression of ATP1A1 and ATP1A3 in human OSC tissues was significantly higher than that in normal ovarian tissues, and that the increase in ATP1A3 expression was significantly correlated with decreased OS and DSS. Conversely, the expression of ATP1A2 and ATP1A4 was higher in normal ovarian tissues than in tumor tissues, and the high expression of ATP1A4 in OSC tissues was significantly correlated with prolonged OS and DSS. In addition, we found that the overexpression of ATP1A2 and ATP1A3 was closely related to poor prognosis of patients with OSC with FIGO stage III, histological grade G3, TP53 mutation, and age ≥ 60 years. However, overexpression of ATP1A4 indicates a relatively good prognosis in these subgroups. Furthermore, we identified that primary therapy outcome, residual tumor, ATP1A3 gene, and ATP1A4 gene were independent indicators of OS and DSS in OSC. Moreover, primary tumor outcome, residual tumor, ATP1A2, ATP1A3, and ATP1A4 were closely related to the risk of recurrence and mortality in OSC. Finally, we conducted preliminary detection of the ATP1A gene family proteins in the tissue samples of six patients with OSC and found that the results were consistent with our prediction. Taken together, these results suggest that abnormal expression and activation of ATP1As play a key role in the occurrence and development of OSC.

To date, the signaling pathways involved in Na^+^/K^+^-ATPase, such as MAPK, ERK, PLC, PKC, and Ras/Raf, have been well understood in normal cells. However, only few studies have focused on these related signaling pathways in tumors [24–28]. Previous studies have reported that Na^+^/K^+^-ATPase can promote tumor multidrug resistance (MDR) by affecting the c-Myc, NF-κB, and *N*-glycosylation pathways [28–30]. Furthermore, ATP1As have been gradually discovered as important therapeutic targets in malignant tumors. Cardiac glycosides are known to be specific inhibitors of Na^+^/K^+^-ATPase enzyme [31], which inhibit enzyme activity by binding to α subunits [32]. Due to the anticancer effects of these drugs found in vitro in certain tumors, they are considered to be promising candidates for cancer treatment [33]. At present, cardiac glycosides or their derivatives have been evaluated in phase I and phase II clinical trials of several refractory and advanced cancers [34–37].

Finally, some limitations in our study warrant discussion. First, when comparing the differential expression of ATP1A2 and ATP1A3 in tumor and normal tissues, it was found that the results from the TCGA and ICGC databases were contradictory. Second, survival analysis based on the TCGA database indicated that a higher expression of ATP1A4 was associated with longer OS and DSS, which is contrary to the results based on the GEO database. These contradictory results might be due to the smaller number of control samples in the OV-AU dataset and the GEO database. Third, the clinical data available in these databases are limited, and data on several important factors such as tumor size, ascites, chemotherapy resistance, and CA125 level, affecting the prognosis of OSC were missing. Finally, we conducted preliminary tests on the expression of ATP1A in limited clinical samples. Although the results are consistent with the predictions, further research is still warranted.

## Conclusions

The present study demonstrated that the ATP1A gene family members are abnormally expressed in OSC tissues and are closely correlated with the clinical features and prognosis of patients with this malignancy. Thus, the expression of ATP1As may be useful biomarkers of the diagnosis and prognosis of OSC. In addition, ATP1As may become effective therapeutic targets for the treatment of OSC.

## Supplementary information

**Additional file 1: Table S1.** Clinical features of OC patients from ICGC. **Table S2.** Clinical features of OC patients from GSE26193.

**Additional file 2: Figure S1.** Expression of ATP1A gene family members in ovarian cancer (OC) and its diagnostic value. (A–D) Differential expression analysis of ATP1A gene family between OC samples and normal ovarian samples based on the GEO database. (E–H) Receiver-operating characteristic (ROC) curves of ATP1A gene family members for diagnosis of OC.

**Additional file 3: Figure S2.** ATP1A gene family member expression in pan-cancers and paracancerous tissues. ATP1A1 (A); ATP1A2 (B); ATP1A3 (C); ATP1A4 (D) (*p < 0.05, **p < 0.01, ***p < 0.001).

**Additional file 4: Figure S3.** Expression of ATP1A gene family members in pan-cancer cell lines. ATP1A1 (A), ATP1A2 (B), ATP1A3 (C), and ATP1A4 (D).

**Additional file 5: Figure S4.** Analysis of ATP1A gene family expression and clinical correlations in ovarian cancer. Correlation between FIGO stage and ATP1A1 (A), ATP1A2 (B), ATP1A3 (C), and ATP1A4 (D). Correlation between histological grade and ATP1A1 (E), ATP1A2 (F), ATP1A3 (G), and ATP1A4 (H).

**Additional file 6: Figure S5.** Prognostic analysis of the ATP1A gene family members in ovarian cancer. (A–D) Effects of ATP1As gene family on overall survival (OS). (E–H) Effects of ATP1A gene family members on progression-free survival (PFS) (*p* < 0.05 was considered statistically significant).

**Additional file 7: Figure S6.** Prognostic analysis of ATP1A gene family members in subgroup for DSS. Prognostic analysis of ATP1A1 (A), ATP1A2 (B), ATP1A3 (C), and ATP1A4 (D) for FIGO stage III. Prognostic analysis of ATP1A1 (E), ATP1A2 (F), ATP1A3 (G), and ATP1A4 (H) for histologic grade G3. Prognostic analysis of ATP1A1 (I), ATP1A2 (J), ATP1A3 (K), and ATP1A4 (L) for TP53 mutation. Prognostic analysis of ATP1A1 (M), ATP1A2 (N), ATP1A3 (O), and ATP1A4 (P) for age ≥ 60 (*p* < 0.05 was considered statistically significant)

## Data Availability

The datasets used and analyzed during the current study are available from the corresponding author on reasonable request.

## Abbreviations

ATP1As: Sodium/potassium adenosine triphosphate (Na^+^/K^+^-ATP)ase α-subunits
CCLE: Cancer Cell Line Encyclopedia
DSS: Disease-specific survival
FIGO: Federation of Gynecology and Obstetrics
GTEx: Genotype-Tissue Expression
HPA: The Human Protein Atlas
OS: Overall survival
OSC: Ovarian serous cystadenocarcinoma
OV-AU: Ovarian cancer-AU
PI3K/AKT: Phosphatidylinositol 3-kinase/protein kinase B
ROC: Receiver operating characteristic
TCGA: The Cancer Genome Atlas
