# Supplementary material for: Comprehensive analysis of the expression of sodium/potassium-ATPase α subunits and prognosis of ovarian serous cystadenocarcinoma
Source: Cancer Cell Int. 2020 Jul 14;20:309. doi: 10.1186/s12935-020-01414-5 (PMC7362554; doi:10.1186/s12935-020-01414-5)
Supplement: Supplementary file 1 — Additional file 1: Table S1. Clinical features of OC patients from ICGC. Table S2. Clinical features of OC patients from GSE26193. [file 12935_2020_1414_MOESM1_ESM.docx]

**Table S1** Clinical features of OC patients from ICGC

| **Characteristics** | **Groups** | **Patients (*N*=81)** | |
| --- | --- | --- | --- |
|  |  | **No.** | **%** |
| Age | Median | 59 |  |
|  | Range | 39-78 |  |
|  | <60 | 43 | 53.09 |
|  | ≥60 | 38 | 46.91 |
| FIGO grade | G2 | 15 | 18.52 |
|  | G3 | 66 | 81.48 |
| Histologic stage | Stage III | 69 | 85.19 |
|  | Stage IV | 12 | 14.81 |

**Table S2** Clinical features of OC patients from GSE26193

| **Characteristics** | **Groups** | **Patients (*N*=107)** | |
| --- | --- | --- | --- |
|  |  | **No.** | **%** |
| FIGO grade | G1-2 | 40 | 37.38 |
|  | G3-4 | 67 | 62.62 |
| Histologic stage | Stage I-II | 31 | 28.97 |
|  | Stage III | 59 | 55.14 |
|  | Stage IV | 17 | 15.89 |
