# Supplementary figures and images for: Comprehensive analysis of the expression of sodium/potassium-ATPase α subunits and prognosis of ovarian serous cystadenocarcinoma
Source: Cancer Cell Int. 2020 Jul 14;20:309. doi: 10.1186/s12935-020-01414-5 (PMC7362554; doi:10.1186/s12935-020-01414-5)

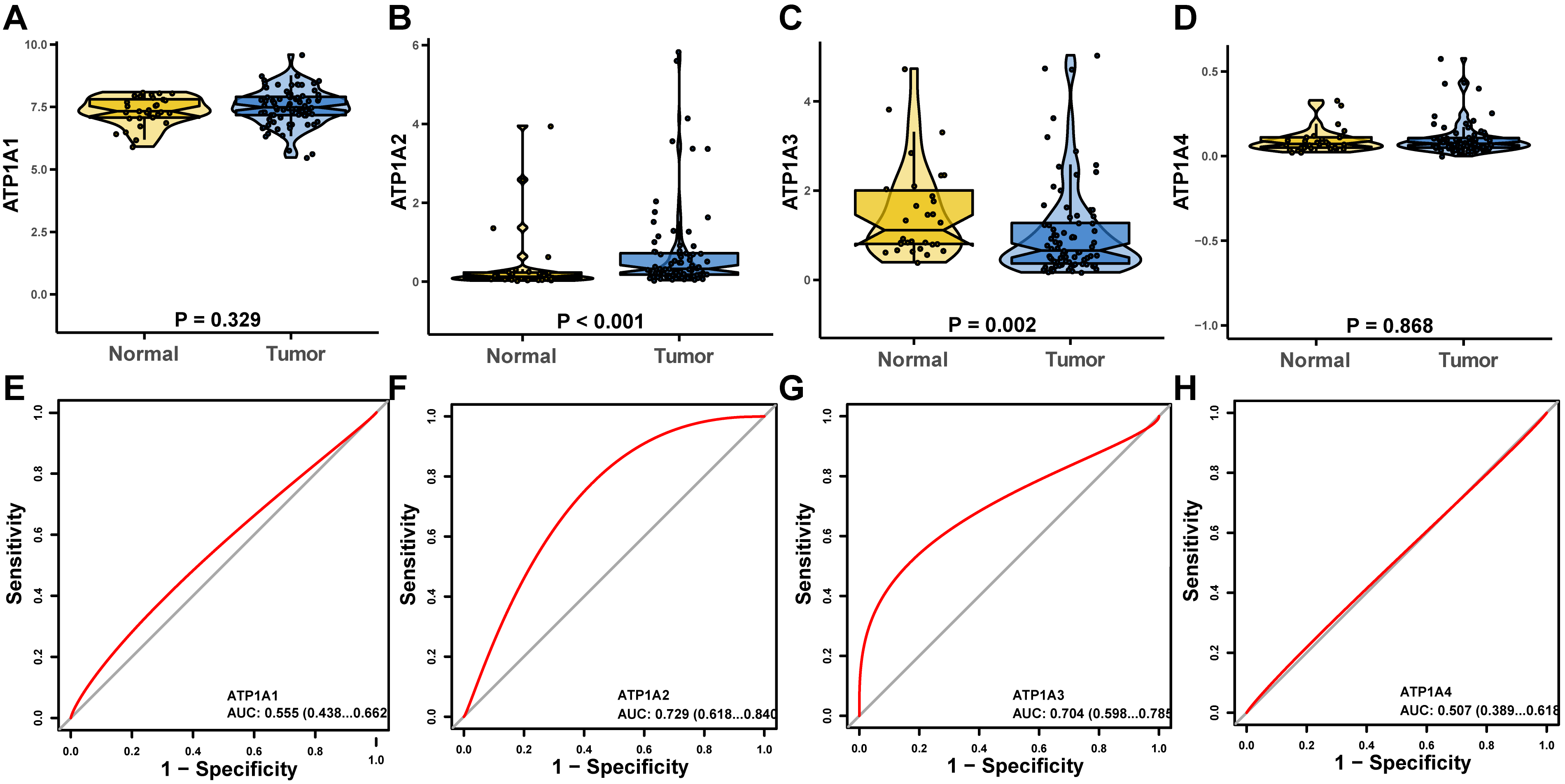

Supplement: Supplementary file 2 — Additional file 2: Figure S1. Expression of ATP1A gene family members in ovarian cancer (OC) and its diagnostic value. (A–D) Differential expression analysis of ATP1A gene family between OC samples and normal ovarian samples based on the GEO database. (E–H) Receiver-operating characteristic (ROC) curves of ATP1A gene family members for diagnosis of OC. [file 12935_2020_1414_MOESM2_ESM.tif]

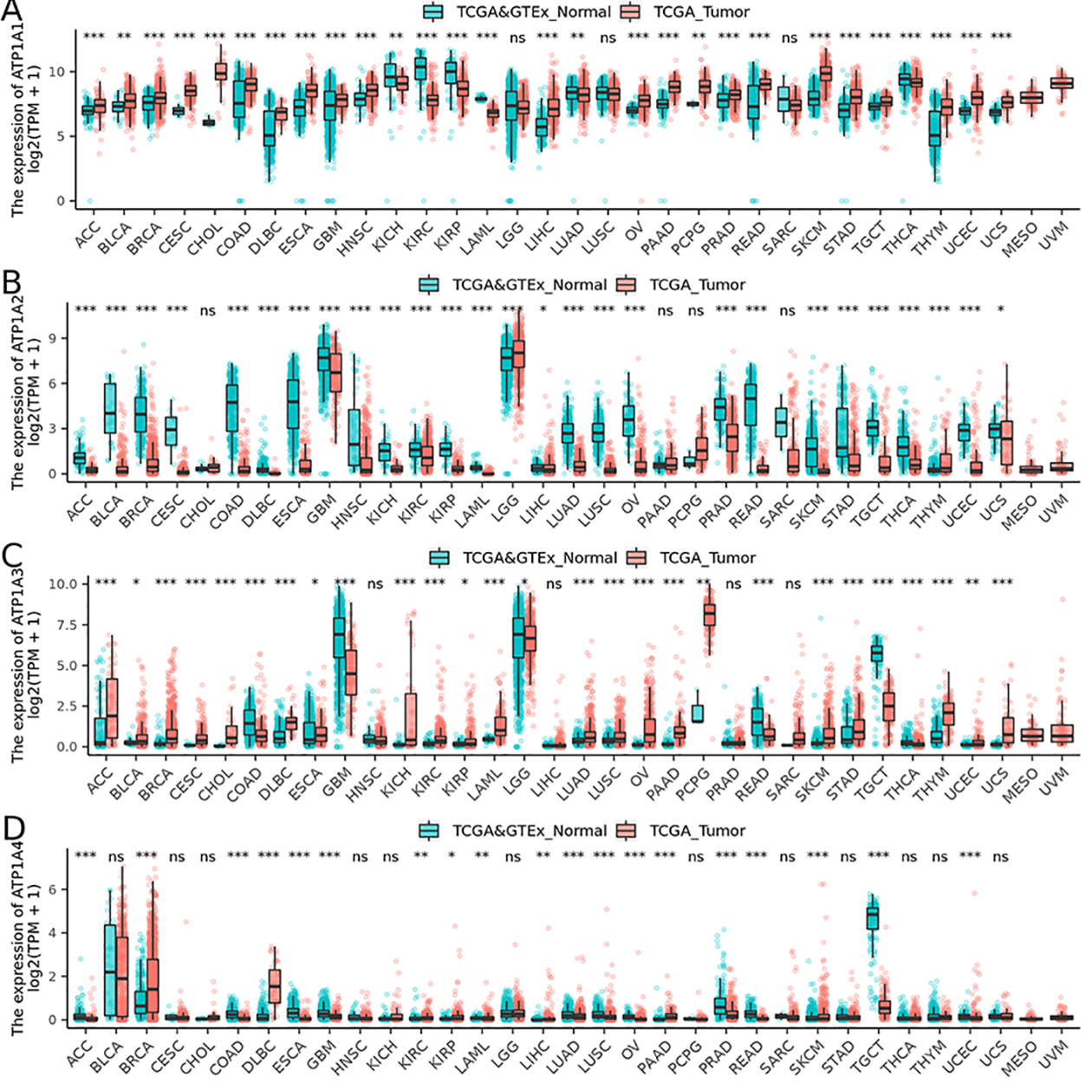

Supplement: Supplementary file 3 — Additional file 3: Figure S2. ATP1A gene family member expression in pan-cancers and paracancerous tissues. ATP1A1 (A); ATP1A2 (B); ATP1A3 (C); ATP1A4 (D) (*p < 0.05, **p < 0.01, ***p < 0.001). [file 12935_2020_1414_MOESM3_ESM.tif]

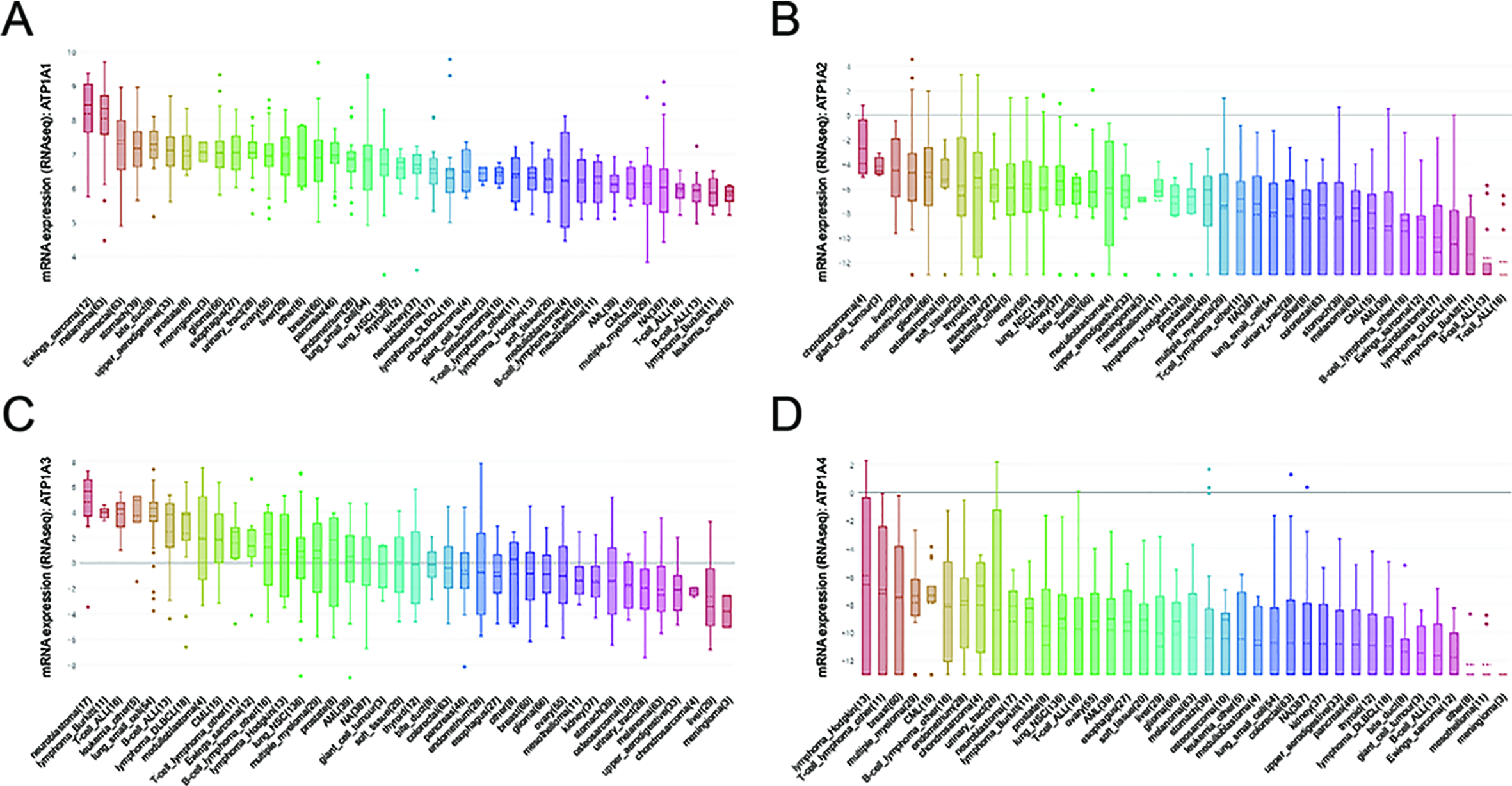

Supplement: Supplementary file 4 — Additional file 4: Figure S3. Expression of ATP1A gene family members in pan-cancer cell lines. ATP1A1 (A), ATP1A2 (B), ATP1A3 (C), and ATP1A4 (D). [file 12935_2020_1414_MOESM4_ESM.tif]

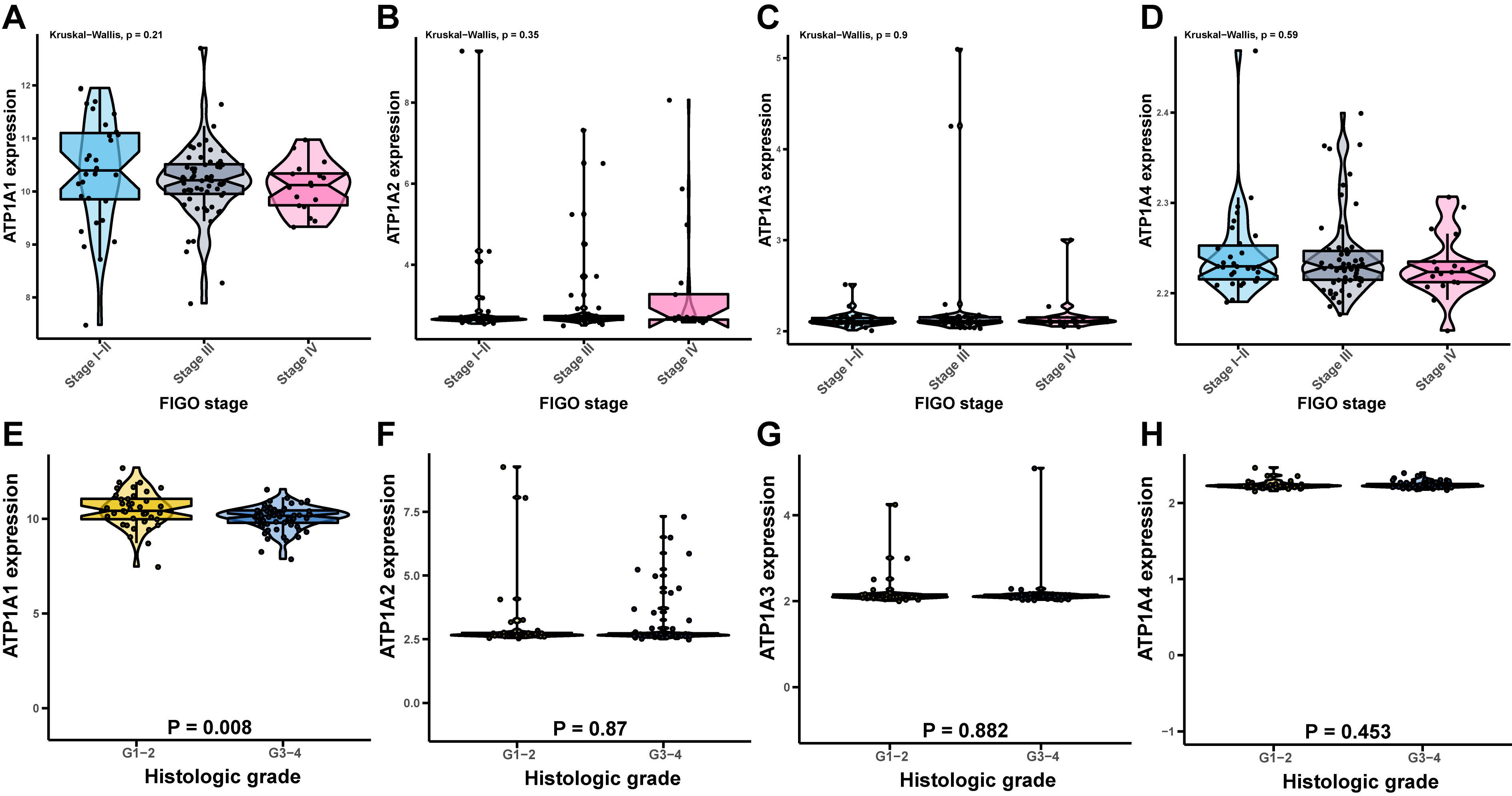

Supplement: Supplementary file 5 — Additional file 5: Figure S4. Analysis of ATP1A gene family expression and clinical correlations in ovarian cancer. Correlation between FIGO stage and ATP1A1 (A), ATP1A2 (B), ATP1A3 (C), and ATP1A4 (D). Correlation between histological grade and ATP1A1 (E), ATP1A2 (F), ATP1A3 (G), and ATP1A4 (H). [file 12935_2020_1414_MOESM5_ESM.tif]

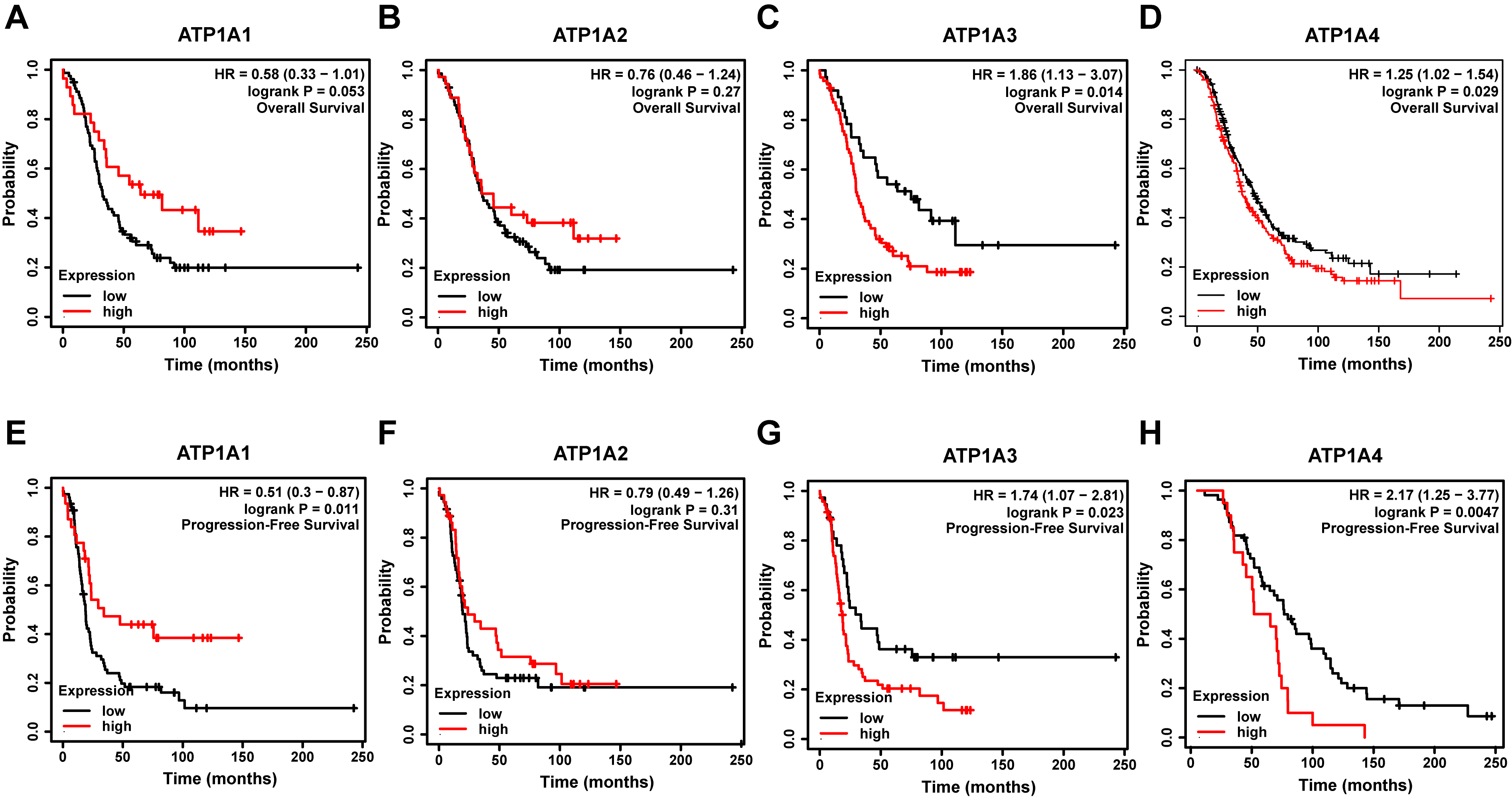

Supplement: Supplementary file 6 — Additional file 6: Figure S5. Prognostic analysis of the ATP1A gene family members in ovarian cancer. (A–D) Effects of ATP1As gene family on overall survival (OS). (E–H) Effects of ATP1A gene family members on progression-free survival (PFS) (p < 0.05 was considered statistically significant). [file 12935_2020_1414_MOESM6_ESM.tif]

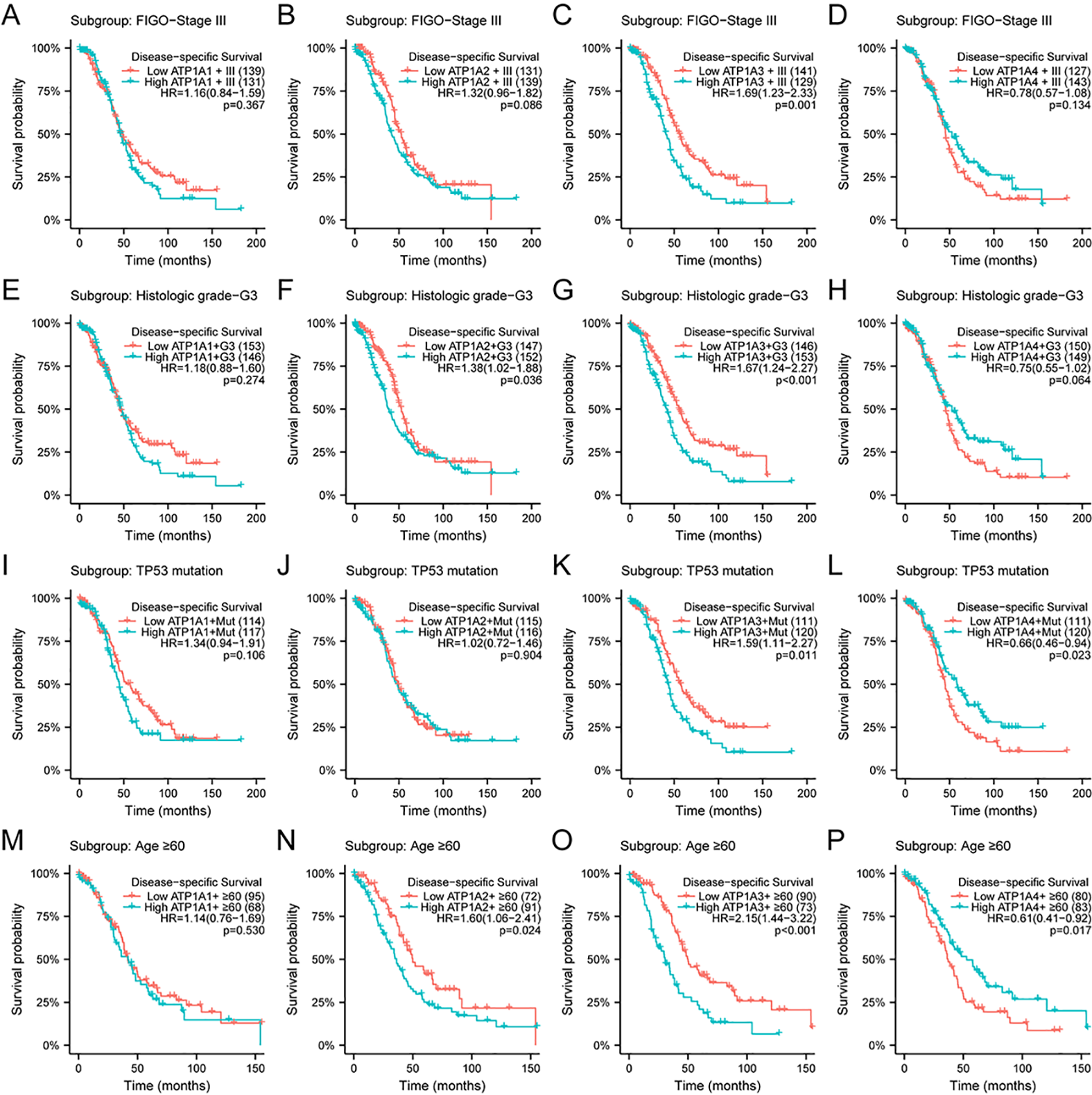

Supplement: Supplementary file 7 — Additional file 7: Figure S6. Prognostic analysis of ATP1A gene family members in subgroup for DSS. Prognostic analysis of ATP1A1 (A), ATP1A2 (B), ATP1A3 (C), and ATP1A4 (D) for FIGO stage III. Prognostic analysis of ATP1A1 (E), ATP1A2 (F), ATP1A3 (G), and ATP1A4 (H) for histologic grade G3. Prognostic analysis of ATP1A1 (I), ATP1A2 (J), ATP1A3 (K), and ATP1A4 (L) for TP53 mutation. Prognostic analysis of ATP1A1 (M), ATP1A2 (N), ATP1A3 (O), and ATP1A4 (P) for age ≥ 60 (p < 0.05 was considered statistically significant) [file 12935_2020_1414_MOESM7_ESM.tif]
